# Cofilin 2 in Serum as a Novel Biomarker for Alzheimer’s Disease in Han Chinese

**DOI:** 10.3389/fnagi.2019.00214

**Published:** 2019-08-09

**Authors:** Yingni Sun, Lisheng Liang, Meili Dong, Cong Li, Zhenzhen Liu, Hongwei Gao

**Affiliations:** ^1^School of Life Sciences, Ludong University, Yantai, China; ^2^Department of Pain, Qingdao University Medical College Affiliated Yantai Yuhuangding Hospital, Yantai, China; ^3^Central Sterile Supply Department, Qingdao University Medical College Affiliated Yantai Yuhuangding Hospital, Yantai, China; ^4^Chemical and Materials Engineering, University of Kentucky, Lexington, KY, United States; ^5^Chemical Engineering and Materials Science, College of Chemistry, Shandong Normal University, Jinan, China

**Keywords:** Alzheimer’s disease, cofilin 2, biomarker, diagnosis, serum

## Abstract

The identification of biomarkers of Alzheimer’s disease (AD) is an important and urgent area of study, not only to aid in the early diagnosis of AD, but also to evaluate potentially new anti-AD drugs. The aim of this study was to explore cofilin 2 in serum as a novel biomarker for AD. The upregulation was observed in AD patients and different AD animal models compared to the controls, as well as in AD cell models. Memantine and donepezil can attenuate the upregulation of cofilin 2 expression in APP/PS1 mice. The serum levels of cofilin 2 in AD or mild cognitive impairment (MCI) patients were significantly higher compared to controls (AD: 167.9 ± 35.3 pg/mL; MCI: 115.9 ± 15.4 pg/mL; Control: 90.5 ± 27.1 pg/mL; *p* < 0.01). A significant correlation between cofilin 2 levels and cognitive decline was observed (*r* = –0.792; *p* < 0.001). The receiver operating characteristic curve (ROC) analysis showed the area under the curve (AUC) of cofilin 2 was 0.957, and the diagnostic accuracy was 80%, with 93% sensitivity and 87% specificity. The optimal cut-off value was 130.4 pg/ml. Our results indicate the possibility of serum cofilin 2 as a novel and non-invasive biomarker for AD. In addition, the expression of cofilin 2 was found to be significantly increased in AD compared to vascular dementia (VaD), and only an increased trend but not significant was detected in VaD compared to the controls. ROC analysis between AD and VaD showed that the AUC was 0.824, which could indicate a role of cofilin 2 as a biomarker in the differential diagnosis between AD and VaD.

## Introduction

Alzheimer’s disease (AD) is the most common fatal neurodegenerative disease of the elderly worldwide (Jagust, 2018). The identification of early biomarkers of AD will allow earlier diagnosis and thus earlier intervention (Wood, 2016; Hampel et al., 2018). New therapeutic strategies in AD are likely to have the best efficacy if they can be implemented early in the disease course (Lansbury, 2004; Livingston et al., 2017).

The pathology of AD is characterized by the progressive loss of basal forebrain cholinergic neurons and by two hallmark features: extracellular senile plaques and intracellular neurofibrillary tangles (NFTs) (Sery et al., 2013). In addition, AD brains show clear signs of oxidative stress along with neuroinflammatory response (Verri et al., 2012). Cytoskeletal abnormalities and synaptic loss are common pathologies in both sporadic and familial AD (Lane et al., 2018). Mild cognitive impairment (MCI) is not a pathological entity but defines a level of decline in cognitive function not interfering with daily activities (Petersen and Negash, 2008; Sanford, 2017).

The diagnosis of AD remain problematic at present (Scheltens et al., 2016; Hampel et al., 2018; Molinuevo et al., 2018). Reduced levels of Aβ, increased total tau (T-tau) or phosphorylated tau (P-tau) in cerebrospinal fluid (CSF) are currently the most promising biomarkers that might predict disease progression (Blennow et al., 2010; Humpel, 2011; Olsson et al., 2016; Polanco et al., 2018). Yet, their changes are not entirely specific to AD (Lleo et al., 2015), and moreover, it is challenging for the combination of Aβ, T-tau or P-tau in CSF to distinguish early AD to controls (Wood, 2016). Some disruption of the blood–brain barrier has been suggested to happen in AD patients, which enables some proteins in CSF to enter into the peripheral blood (Sweeney et al., 2018). Thus, blood biomarkers could reflect the alterations in brains. Not surprisingly, the blood biomarkers were focused on Aβ, T-tau and P-tau, but it turned out to be disappointing (O’Bryant et al., 2015; Hampel et al., 2018). In recent years, many candidate proteins in blood have been found using the proteomic approach (Lista et al., 2013; Robinson et al., 2017; Hondius et al., 2018), but the following validation research was often ignored, or the results were unsatisfactory. Evidences suggest that miRNAs have great potential for use as a biomarker in AD and other neurodegenerative disorders (Bekris and Leverenz, 2015; Pan et al., 2016). However, challenges still exist due to the lack of extensive validation and follow-up in larger cohorts of patients, and miRNAs are not yet a viable diagnostic or therapeutic tool for AD (Martinez and Peplow, 2019).

Cofilin as the major ADF/cofilin isoform in mammalian neurons influences the dynamics of actin assembly by severing or stabilizing actin filaments (Shaw and Bamburg, 2017). Cofilin is crucial for normal structure, dynamics, and function of the cytoskeleton, thus abnormalities in this protein can cause significant cytoskeletal disruption (Maloney and Bamburg, 2007; Bernstein and Bamburg, 2010; Bravo-Cordero et al., 2013). Because cofilin plays the central role in the regulation of actin filaments dynamics, it is involved in the development of neurodegenerative diseases, cancer and cardiomyopathies (Wang et al., 2007; Bamburg et al., 2010; Bravo-Cordero et al., 2013; Schonhofen et al., 2014; Chang et al., 2015; Subramanian et al., 2015; Bamburg and Bernstein, 2016). Three isoforms are known: cofilin 1, destrin and cofilin 2 (Bamburg and Wiggan, 2002; Shishkin et al., 2016). Cofilin 1 was mainly presented in non-muscle cells and in embryonic muscle cells (Shishkin et al., 2016). Destrin was expressed primarily in epithelial and endothelial cells. Cofilin 2 has two isoforms, CFL2a and CFL2b (Ostrowska and Moraczewska, 2017). The former is expressed in a wide variety of tissues, whereas the latter is expressed predominantly in skeletal and cardiac muscle (Shishkin et al., 2016). The protein cofilin 2 is composed of 5 α helices, 5 β sheets, and 1 C-terminal β short chain, with a molecular weight of 18 kDa (Ostrowska and Moraczewska, 2017). Studies of cofilin pathology have helped explain the development of sporadic (late onset) AD and have furthered our understanding of familial AD (Maloney and Bamburg, 2007; Zempel et al., 2017; Borovac et al., 2018; Rush et al., 2018). However, little was known whether cofilin can act as a biomarker of AD. In addition, most of above studies involved in AD either did not differentiate cofilin 1 from cofilin 2 (Whiteman et al., 2009; Rahman et al., 2014; Woo et al., 2015a; Deng et al., 2016; Shaw and Bamburg, 2017), or have only focused on cofilin 1 (Barone et al., 2014; Rush et al., 2018), but only few has concerned about cofilin 2 in AD.

Our previous proteomics study showed that the protein level of cofilin 2 was elevated greatly in the hippocampus of APP/PS1 transgenic mice compared with wild type (WT) mice, as well as in small amounts of AD serum samples (Sun et al., 2015). In the present study, we validated this result in a larger population, and moreover, we analyzed the expression of cofilin 2 in different AD animal and cell models. The protein expression and phosphorylation of cofilin 2 in the hippocampus tissues from AD patients and controls were also evaluated. To test whether cofilin 2 could be proposed as a non-invasive biomarker of AD, we compared the serum levels of cofilin 2 in AD, MCI patients and controls by enzyme-linked immunosorbent assay (ELISA), and the diagnostic accuracy as a biomarker was evaluated through the receiver operator characteristic curve (ROC) analysis. The correlation analysis between cofilin 2 levels and cognitive decline was also performed to determine whether higher cofilin 2 expression was associated with more severe disease or not. Additionally, to test whether cofilin 2 could distinguish AD from another common dementia-vascular dementia (VaD), we measured cofilin 2 serum levels in VaD, compared the expressions between AD and VaD, and also performed the relevant ROC analysis.

## Materials and Methods

### Control and AD Brains

Frozen hippocampal samples of 10 AD patients and 10 age-matched controls were obtained from the Brain Bank of Chinese Academy of Medical Sciences and Peking Union Medical College, which collects brains from donors through a whole-body donation program. All procedures were approved by the Institutional Review Board (IRB). All AD patients displayed progressive intellectual decline and met NINCDS-ADRDA Workgroup criteria for the clinical diagnosis (McKhann et al., 1984). All controls had test scores in the normal range. The written informed consents for using the donated body tissue were given all donors for medical search. After death, the bodies were transferred rapidly to a designated autopsy facility. The average postmortem intervals (PMIs) were less than 3 h. Brains were bisected along the sagittal plane. One half was fixed in 10% phosphate-buffered formaldehyde, the other was cut into coronal slices (1 cm), and stored in –80°C until ready to use. These fixed hemi-brain blocks were sampled systematically, paraffin-embedded, and processed for standard immunohistologic and histologic stains as recommended (Montine et al., 2012). Hematoxylin-eosin and modified-Bielschowsky staining, Aβ antibody (10D5), and α-synuclein immunohistochemistry were used for diagnosis on multiple neocortical, hippocampal, cerebellum and entorhinal sections.

### Human Serum Sample Collection and Preparation

A total of 181 AD subjects, 58 MCI subjects and 181 non-demented healthy controls matched for age and gender were recruited in Yuhuangding Hospital for this study. Detailed demographic information of the subjects enrolled in the study is presented in Table 1.

**TABLE 1:** Demographics characteristics of the study samples.

	**Control** **(*n* =** **181)**	**MCI** **(*n* =** **58)**	**AD** **(*n* =** **181)**	**ANOVA** ***p*-value**
Age, years	73.5 ± 5.1	72.7 ± 5.9	74.2 ± 6.3	N.S.
Male patients	80 (44.2%)	27 (46.6%)	85 (47.0%)	N.S.
BMI	27.2 ± 4.6	26.0 ± (3.8	26.8 ± 4.5	N.S.
MMSE, points	28.8 ± 0.7	26.0 ± 2.9	16.8 ± 4.6	< 0.001
Education, years	9.4 ± 3.1	8.8 ± 2.8	9.6 ± 3.2	N.S.

*Data presented as mean ± SD where appropriate. *Post hoc* Tukey test: MMSE: AD vs. Control, *p* < 0.001; AD vs. MCI, *p* < 0.001; MCI vs. Control, *p* < 0.001. N.S. not significant different. MCI, mild cognitive impairment; AD, Alzheimer’s disease; BMI, body mass index; MMSE, mini-mental status examination.*

All the subjects underwent a standard set of evaluations including past medical history review, laboratory tests, neurologic examinations and brief neuropsychological assessments (Fernandez Montenegro and Argyriou, 2017; Vallejo et al., 2017). Cases had a clinical diagnosis of probable AD according to DSW-IV, ICD-10 and NINCDS-ADRDA criteria. The cognitive status and severity of dementia were assessed by the Mini-Mental State Exam (MMSE) and the Clinical Dementia Rating (CDR) testing. Controls had no signs suggesting cognitive decline, and had a MMSE score between 28 and 30 and a CDR score of zero. Controls were excluded if they presented or had a history of depression or psychosis, substance abuse, or use of medications that could impair cognitive function. All controls were followed clinically for 2 years in order to rule out the development of cognitive decline. AD patients were followed-up and their cognitive status was reassessed 6 months after enrollment. MCI subjects were otherwise healthy, without significant medical, neurological, or psychiatric disease and met the following Petersen’s criteria: (i) memory complaints by participant or family; (ii) objective signs of decline in any cognitive domain; (iii) normal activities of daily living; and (iv) the clinical features do not satisfy the DSM-IV/ICD-10 criteria (Petersen et al., 1999). In this study, no subject, originating from Northern Han Chinese populations, was presented with major and known co-morbidities, including hypertension, cardiopathy, diabetes or renal dysfunction.

This study also included 32 patients with VaD whose diagnoses were confirmed using NINDS-AIREN criteria (Roman et al., 1993). They are matched with the control group in age of onset, gender, body mass index (BMI) and educational level. Written informed consents were acquired from all subjects. The protocol of the study was approved by the Institute Ethical Committee of the Affiliated Yuhuangding Hospital of Qingdao University.

Blood samples (5 ml) were drawn in the morning hours under standardized conditions after an overnight fasting period. Blood was collected in evacuated collection tubes without anticoagulant and allowed to clot for 2 h on ice prior to centrifugation at 4,000 *g* for 8 min at 4°C. Serum was aliquotted (50 μl/tube) and stored in Eppendorf tubes at −80°C until utilized.

### AD Animal Models

Groups of APP/PS1 double transgenic mice and age-matched WT mice (*n* = 8–10 per group) were purchased from the Jackson Laboratory Company [strain name B6C3-Tg (APPswe, PSEN1dE9) 85Dbo/J; stock number 004499]. Memantine and Donepezil were purchased from Sigma-Aldrich. They were dissolved respectively in distilled water. APP/PS1 transgenic mice were randomly divided into three groups of 8–10 mice each: untreated APP/PS1 Tg model, Memantine (Tg + 30 mg/kg Memantine) and Donepezil (Tg + 30 mg/kg Donepezil) groups. WT and untreated Tg model groups received distilled water alone. The administration by oral gavage was started at 12 months old and lasted for 12 weeks.

Aβ oligomers were prepared according to the protocols published by our group (Li et al., 2014). Synthetic Aβ_25–35_ was purchased from Sigma. The concentration of Aβ_25–35_ depends on the volume of the rat CSF. Male Wistar rats (3 months old, 220–250 g) were obtained from the Experimental Animal Center of Ludong University. 1 nM Aβ_25–35_ was injected into the lateral cerebral ventricle of these rats.

All the mice and rats were kept in a temperature-controlled room at 25°C under a 12-h light/dark cycle and provided water and a commercial pelleted feed *ad libitum*. All the experiments were approved in according to the institutional guidelines of the Experimental Animal Center of Ludong University.

### Cell Culture

SK-N-SH/SK-N-SH APP_695_ human neuroblastoma cells were cultured using Dulbecco’s modified Eagle’s medium (DMEM) culture in supplement with 10% fetal bovine serum, 100 U/ml penicillin and 100 μg/ml streptomycin, and maintained in a humidified atmosphere containing 5% CO_2_ at 37°C. In addition, the SK-N-SH APP_695_ cells were supplemented with G418 (200 μg/ml). Cells were grown until nearly confluent, and then were collected.

The primary rat hippocampal neurons were separated from the brains of embryonic 18–19 (E18-19) Sprague-Dawley. Rat fetuses were dissociated for 20 min both enzymatically (0.25% trypsin-EDTA) and mechanically before filtering through a 100 μm cell strainer. The cell suspension was diluted in high glucose DMEM, 5% horse serum, 10% FBS, and 2 mM L-glutamine, and then plated into 6-well plates coated with poly-D-lysine (20 μg/ml) with the cell density of 1 × 10^5^ cells/ml. Cells were maintained in a humidified atmosphere containing 5% CO_2_ at 37°C. To inhibit the growth of glial cells, the medium was replaced by serum-free neurobasal medium containing supplement B27 and L-glutamine (0.5 mM) after almost 20 h. The half of the culture medium was changed every 3 days. Cells were incubated with 10, 30, and 100 μM Aβ_25–35_ for 48 h separately, which was dissolved in distilled water for 7 days at 37°C before use. Cell culture reagents were obtained from Invitrogen, whereas all other reagents were purchased from Sigma.

### Western Blot Analysis

Standard western blot analysis was carried out. All these mice and rats were sacrificed by CO_2_ inhalation after behavioral testing was completed. The brains were removed and hippocampus was dissected on ice, and then were homogenized thoroughly in a RIPA lysis buffer [150 mM NaCl, 50 mM Tris (pH 7.4), 1% NP40, 0.5% sodium deoxycholate and 0.1% SDS]. The blood samples from mice and rats were collected into the evacuated collection tubes without anticoagulant, and treated in a similar manner with the human serum samples. Tissue sections from frozen hippocampus regions of AD patients and controls were dissected and resuspended in the above lysis buffer. These hippocampal samples were ultrasonicated for 1 min in cycles of 3 s on and 3 s off using a Fisher 550 Sonic Dismembrator. Then the samples were centrifuged at 20,000 *g* at 4°C for 60 min to remove the debris. The supernatants were collected and stored at –80°C before use.

All the cells were collected, and then total protein was extracted in the following lysis buffer containing 150 mM NaCl, 10 mM Tris, 10% glycerol, 1% NP40, 10 mM NaF, 1 mM Na_3_VO_4_, 1 mM EGTA and complete protease inhibitor. Then, the homogenate was centrifuged at 16,000 *g* at 4°C for 20 min. The protein solutions were collected and stored at –80°C before use. Protein concentration was measured with a BCA kit.

All the samples were subjected to electrophoresis, transferred onto PVDF membranes and incubated with the primary antibodies: rabbit anti-cofilin 2 (1:500, Cell Signaling Technology), rabbit anti-cofilin 2 (phospho S3) (1:1000, Abcam), mouse anti-β-actin (1:10000, Sigma) and mouse anti-IgG (1:10000, Abcam). Specifically, equal amounts of protein (40 μg) were run on 10% polyacrylamide gel, transferred on to PVDF membrance, blocked with 5% fat-free milk in Tris-Buffered Saline with Tween-20 (TBST) for 1 h, and subsequently incubated with primary antibody overnight. After washing with TBST for 5 times, the membranes were incubated with horseradish peroxidase (HRP)-coupled secondary antibody (1:10000, Cell Signaling Technology) at room temperature for 1 h with gentle agitation. Finally, membranes were revealed with the ECL Plus kit and High Performance Chemiluminescence Films (GE Healthcare, United States). Digital images of western blots were obtained with the LAS4000 FujiFilm imaging system (FujiFilm, Japan). The densitometric analysis was made by Quantity-One software (Bio-Rad, United States). The values were normalized to β-actin intensity levels.

### Cofilin 2 ELISA

Serum cofilin 2 levels were detected using a commercially available human cofilin 2 quantitative sandwich enzyme immunoassay (Uscnk, Wuhan, China) according to the manufacturer’s instructions. This kit was based on sandwich enzyme-linked immune-sorbent assay technology. Anti-cofilin 2 antibody was pre-coated onto 96-well plates. One hundred microliter of the standards and test samples were pipetted into the wells and were incubated for 2 h at 37°C subsequently. Any cofilin 2 present was bound by the immobilized antibody. The liquid of each well was removed without washing. After that, 100 μl of biotin-conjugated antibody specific for cofilin 2 was added to each well and incubated for 1 h at 37°C. During the incubation, biotin-antibody may appear cloudy. Then, they were warmed up to room temperature and mixed gently until solution appears uniform. Each well was aspirated and washed with wash buffer (200 μl) for three times. After the washing, the avidin conjugated HRP (100 μl) was added to each well and incubated for 1 h at 37°C. The aspiration/wash process was repeated for five times to remove any unbound avidin-enzyme reagent. TMB substrate (90 μl) was added to each well and incubated for 15–30 min at 37°C. After that, the stop solution (50 μl) was added to each well, and the optical density within 5 min was determined using a MQX200 microplate reader (Bio-Tek, United States) set to 450 nm. The serum level of cofilin 2 in the samples was interpolated from kit-specific standard curves generated using GraphPad Prism software.

Intra-assay Precision (Precision within an assay): CV% < 8%. Inter-assay Precision (Precision between assays): CV% < 10%. Three samples of known concentration were tested twenty times on one plate to assess. The detection range of the ELISA kit is 15.6–1000 pg/ml. The minimum detectable dose of human cofilin 2 is less than 3.9 pg/ml. The sensitivity of this assay, or Lower Limit of Detection (LLD) was defined as the lowest protein concentration that could be differentiated from zero. It was determined the mean OD value of 20 replicates of the zero standard added by their three standard deviations. This assay has high sensitivity and excellent specificity for detection of human cofilin 2. No significant cross-reactivity or interference between human cofilin 2 and analogs was observed.

### Statistical Analysis

The data was analyzed using SPSS 13.0 software. Comparison between the groups was made using Student’s *t*-test and one-way ANOVA. Correlations between cofilin 2 level and MMSE scores were performed with the Spearman correlation coefficient. Sensitivity and specificity of the measured variable for AD diagnosis were determined by ROC analysis. The best cut-off value was selected as those which minimize the sensitivity-specificity difference and maximize discriminating power of the tests. Statistical significance was set at *p* < 0.05.

## Results

### Increased Cofilin 2 Expressions in Different AD Animal and Cell Models

**FIGURE 1:**
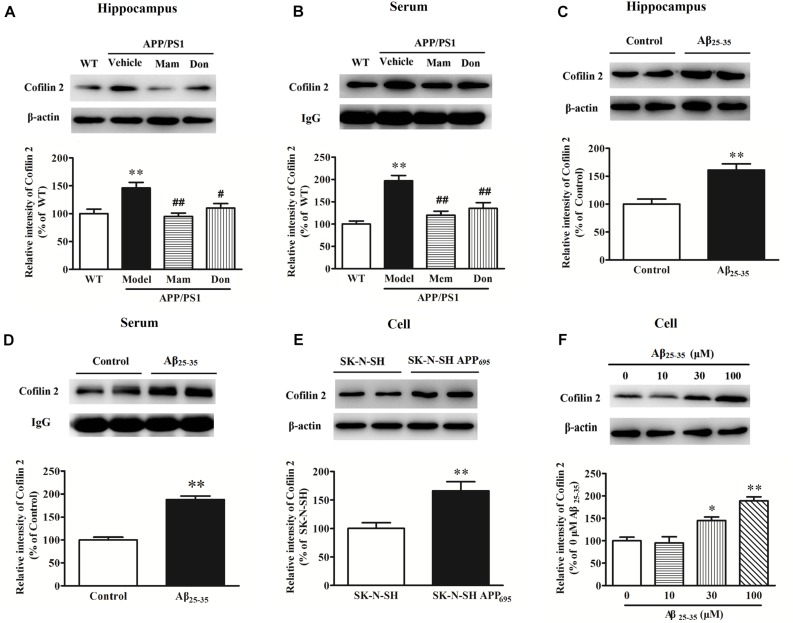
Western blot analysis of cofilin 2 in different animal and cell models. **(A)** Cofilin 2 expression in the hippocampal brain homogenates of 12-month old APP/PS1 and WT mice and the effect of Memantine and Donepezil on cofilin 2. **(B)** Cofilin 2 levels in serum samples from 12-month old APP/PS1 and WT mice and the effect of Memantine and Donepezil on cofilin 2. **(C)** Cofilin 2 expression in the hippocampus of Aβ_25–35_ intracerebroventricular-injected rats. **(D)** Cofilin 2 levels in serum samples from Aβ_25–35_ intracerebroventricular-injected rats. **(E)** Cofilin 2 expression in the SK-N-SH APP_695_ cells. **(F)** Cofilin 2 expression in the primary-cultured hippocampal neurons from rats incubated with Aβ_25–35_. Quantified results were normalized to β-actin/IgG expression. For all the results above, a representative experiment of three performed is shown. Values were expressed as percentages compared to the WT group (set to 100%), and represented as group mean ± SEM. *n* = 8–10 per group. ^*^p < 0.05, ^∗∗^*p* < 0.01 vs. control group; ^#^*p* < 0.05, ^##^*p* < 0.01 vs. untreated APP/PS1 mice.

Western blot analysis was performed to validate changes in protein expressions for cofilin 2 in different AD animal and cell models. As shown in Figure 1A, cofilin 2 was significantly increased in the hippocampus of APP/PS1 mice compared with WT mice, consistent with our previous report (Sun et al., 2015). Similarly, a significant upregulation of cofilin 2 was observed in serum samples from APP/PS1 mice (Figure 1B). In order to observe the effects of positive anti-AD drugs on cofilin 2 expressions, APP/PS1 mice were orally administrated Memantine and Donepezil, respectively. After the long-term treatment, the expression of cofilin 2 was measured by western blot. Quantitative analysis exhibited that the increases of cofilin 2 in the hippocampus and serum samples were significantly attenuated with the treatment of Memantine or Donepezil (Figures 1A,B). In addition, we assessed the expression of cofilin 2 in Aβ_25–35_ intracerebroventricular-injected rat AD model, and found that cofilin 2 was obviously increased by 61% in the hippocampus and by 88% in serum compared to the control rats (Figures 1C,D).

At the meantime, cofilin 2 expression was also detected in AD cell models. As shown in Figures 1E,F, cofilin 2 was increased significantly by 1.7-fold in SK-N-SH APP_695_ cells, and by 1.5/1.9-fold in 30/100 μM Aβ_25–35_-treated primary-cultured hippocampal neurons from rats compared to control group. All these indicated that cofilin 2 was likely to be closely linked with AD pathology.

### Increased Cofilin 2 in the Hippocampus Tissues of AD Patients

**FIGURE 2:**
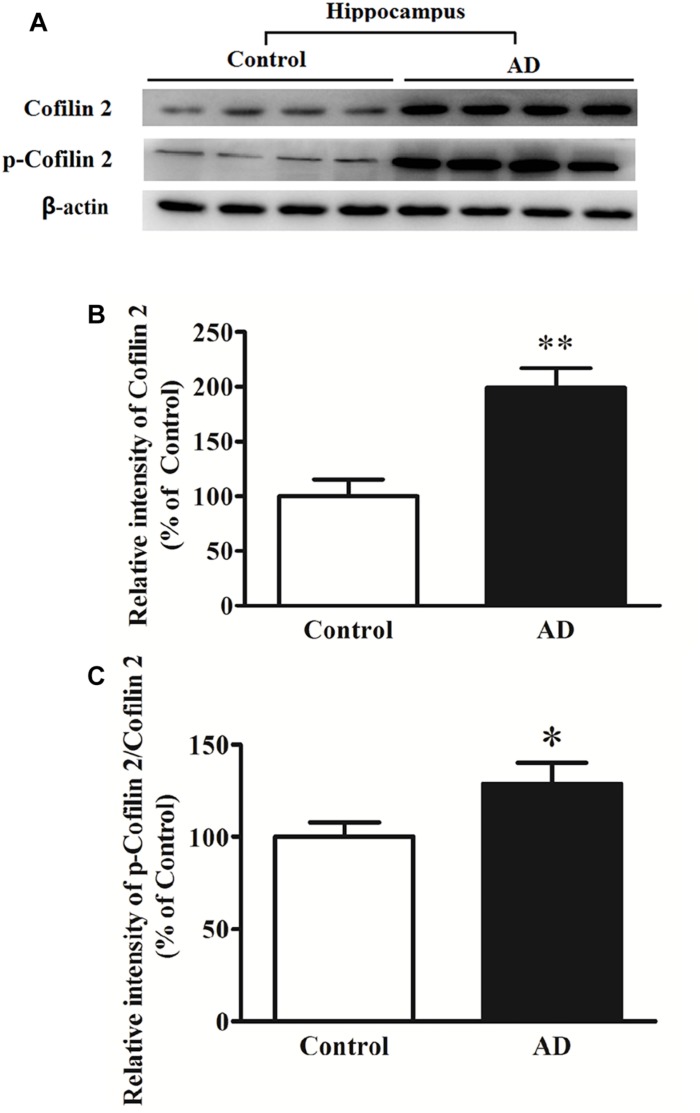
Western blot analysis of cofilin 2 and phospho-cofilin 2 in the hippocampus tissues from AD patients and controls. **(A)** Representative western blot bands. **(B)** Quantitative analysis of cofilin 2 expression. **(C)** Quantitative analysis of cofilin 2 phosphorylation. Quantified results were normalized to β-actin expression. Values were expressed as percentages compared to the controls (set to 100%), and represented as mean ± SEM. *n* = 10 per group. ^*^p < 0.05, ^∗∗^*p* < 0.01 vs. the control group, Student’s *t*-test.

In different AD animal and cell models, cofilin 2 was validated to be increased significantly. To determine whether cofilin 2 was also upregulated in brain tissues of AD patients, we detected the expression of cofilin 2 in the hippocampal sections from AD patients and controls after death (Figure 2A). Meanwhile, the phosphorylation of cofilin 2 was also assessed (Figure 2A). The activity of cofilin 2 is regulated by reversible phosphorylation on ser3, rendering it inactive. Western blot analysis showed a statistically significant increase in protein expressions by 99% (Figure 2B), and by 29% in phosphorylation levels of cofilin 2 in AD samples (Figure 2C).

### Serum Cofilin 2 Levels Detected by ELISA in AD, MCI and Controls

Results were validated subsequently by ELISA in a large population. All samples were comparable in terms of age, education and gender distribution. As expected, AD patients had a lower MMSE score than the healthy controls (mean MMSE score: 28.8 ± 0.7 versus 16.8 ± 4.6) (Table 1).

**FIGURE 3:**
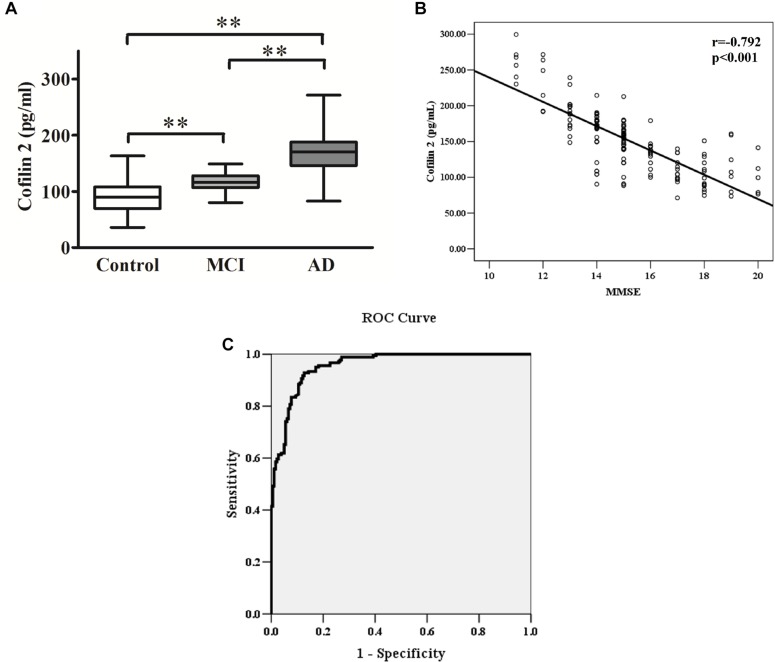
**(A)** Cofilin 2 levels in serum are presented as box plots for AD (*n* = 181), MCI (*n* = 58) and healthy controls (*n* = 181). The lower and upper sides of the boxes indicate the 25th and 75th percentiles, and the horizontal lines indicate the means. Shown are also the lower and upper whiskers that indicate the minimum and maximum values, respectively. ^∗∗^*p* < 0.01 from one way ANOVA with Tukey–Kramer method as a *post hoc* test. **(B)** Correlation analysis between cofilin 2 serum levels and MMSE scores in AD patients. Correlation was assessed using the Spearman correlation coefficient. The concentration of serum cofilin 2 is plotted against MMSE scores for each patient. A significant negative correlation between serum cofilin 2 level and MMSE score (*r* = –0.792, *p* < 0.001) was observed. Correlation lines are also shown. **(C)** ROC curve analysis for serum cofilin 2 concentration and the prediction of the presence of AD. The AUC was 0.957. The optimal cut-off value (130.4 pg/ml) was selected. The diagnostic accuracy for cofilin-2 protein levels was 80% with the sensitivity and specificity 93 and 87%, respectively.

The ELISA results showed that AD patients presented higher serum levels of cofilin 2 in comparison to the controls, and cofilin 2 in MCI group was significantly higher than the control group and significantly lower than the AD group (AD: 167.9 ± 35.3 pg/ml, MCI: 115.9 ± 15.4 pg/ml, Control: 90.5 ± 27.1 pg/ml, *p* < 0.01). The 95% confidence intervals (CIs) were 162.7–173.1 pg/ml in AD, 111.9–119.9 pg/ml in MCI, and 86.5–94.5 pg/ml in Control. The results showed no overlap of 95% CIs among AD, MCI and Control groups for cofilin 2, indicating the changes were statistically significant. Corresponding results are shown in Table 2 and Figure 3A.

**TABLE 2:** Protein levels of cofilin 2 in serum of AD patients, MCI and controls.

	**Control**	**MCI**	**AD**
Protein Level (pg/ml)	90.5 ± 27.1	115.9 ± 15.4^∗∗^	167.9 ± 35.3^∗∗^ **^##^**
The 95% CIs	86.5–94.5	111.9–119.9	162.7–173.1

^*^^*^p-value < 0.01 vs. Control, **^##^***p*-value < 0.01 vs. MCI. One way ANOVA followed by Tukey–Kramer test. Data are means ± SD. *n* = 181 for Control and AD, and 58 for MCI. CIs, confidence intervals.

### Correlation Analysis Between Cofilin 2 Serum Level and Cognitive Decline in AD Patients

The MMSE score is an important measure of the cognitive level of AD patients. The correlation between cofilin 2 serum levels and MMSE scores of patients is shown in Figure 3B. The results showed a significant negative correlation between cofilin 2 serum level and the cognition (evaluated by MMSE scores) within the AD group (*r* = −0.792, *p* < 0.001).

### ROC Curve Analysis

To evaluate the diagnostic value of serum cofilin 2 as a potential biomarker of AD, ROC curve analysis of the ELISA results from AD and control groups was performed. As shown in Figure 3C, the area under the curve (AUC) was 0.957. The optimal cut-off value of 130.4 pg/ml was selected with sensitivity, specificity and diagnostic accuracy for serum cofilin 2 of 93, 87, and 80%, respectively, which could differentiate AD patients from controls.

### Increased Serum Cofilin 2 Levels in AD Compared to VaD

There are many types of dementia that can be difficult to differentiate based on clinical features alone, despite vastly different underlying pathology. AD is characterized by the accumulation of Aβ peptides and hyperphosphorylated Tau (Hoppe et al., 2015; Bourdenx et al., 2017), whereas VaD is caused by the occurrence of many minor ischemic strokes over time (Ray et al., 2013). Other types of dementia include Lewy body dementia (LBD), frontotemporal dementia (FTD), multiple system atrophy dementia (MSA-D) and Parkinson’s disease dementia (PDD), which also each have their own unique pathology. AD and VaD are the most common types (Posada-Duque et al., 2014). However, it can be challenging to differentiate them based on the clinical features alone.

**FIGURE 4:**
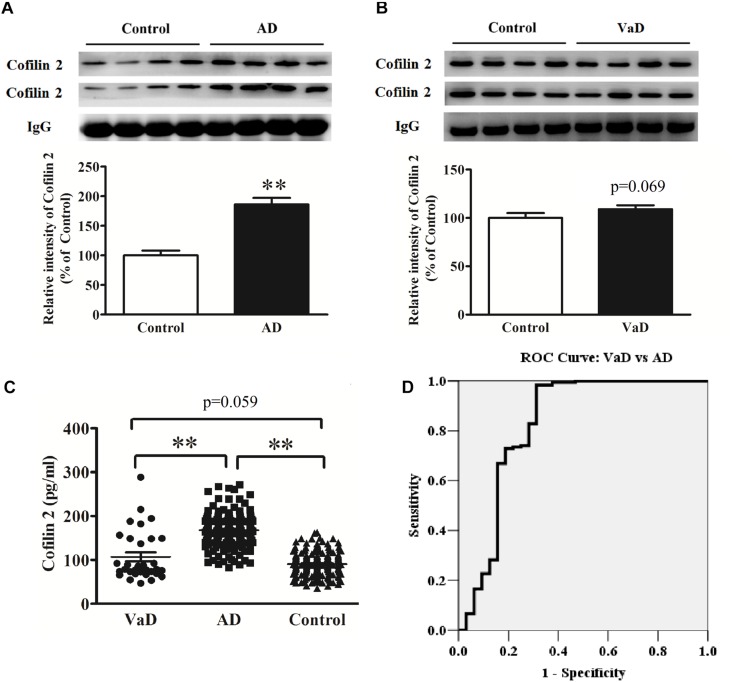
Upregulation of serum cofilin 2 in AD samples **(A)**, and increased trends without significant difference in VaD samples compared to the controls **(B)**. The individual sera samples corresponding to AD/VaD and controls were analyzed by western blots for cofilin 2 protein expression. Equal volume of each sample was loaded and IgG was used as a loading control. Values were expressed as percentages compared to control group (set to 100%), and represented as group mean ± SEM. *n* = 32 per group. ^∗∗^*p* < 0.01 versus the control group, Student’s *t*-test. **(C)** Scatter plots of cofilin 2 levels in serum of VaD (*n* = 32), AD (*n* = 181) and Controls (*n* = 181) measured by ELISA. Protein expression of cofilin 2 was significantly increased in AD compared to VaD. Data represent means ± SEM. ^∗∗^*p* < 0.01. **(D)** ROC curve analysis for serum cofilin 2 between VaD and AD. The AUC was 0.824.

Therefore, we detected cofilin 2 levels in VaD serum. Western blot analysis showed that cofilin 2 was obviously enhanced in serum of AD patients but only had an increased trend but not significantly in VaD patients compared to the control group (Figures 4A,B). ELISA analysis showed the similar results to the western blot, and the serum level of cofilin 2 in VaD was detected to be 107.1 ± 57.1 pg/mL (Figure 4C). It was also shown that cofilin 2 was significantly increased in AD compared to VaD in Figure 4C. ROC curve analysis of the levels of cofilin 2 between AD and VaD showed that AUC was 0.824 (Figure 4D), suggesting that cofilin 2 might act as a marker that could distinguish AD from VaD.

## Discussion

This study explored the potential of cofilin 2 as a candidate biomarker for AD. Our results indicated that cofilin 2 expression was significantly higher in different AD animal and cell models, as well as in AD patients. Memantine as *N*-methyl-D-aspartate (NMDA) receptor antagonist and Donepezil as acetylcholinesterase inhibitors, are currently effective drugs for AD (Standridge, 2004; Chen et al., 2017; Graham et al., 2017). In this study, the upregulated cofilin 2 was significantly attenuated after the treatment with Memantine and Donepezil in APP/PS1 mice, indicating that cofilin 2 might remain closely tied to the pathology of AD.

Many previous studies have demonstrated that cofilin may contribute to AD pathogenesis (Bamburg et al., 2010; Bamburg and Bernstein, 2016; Shaw and Bamburg, 2017; Rush et al., 2018). Cofilin and actin can form rod-like structures within neurites of AD brain (Minamide et al., 2000), and their dysfunction may mediate the loss of synapses, and production of the hallmark pathological features of AD: excess Aβ and NFTs (Maloney and Bamburg, 2007; Bamburg and Bernstein, 2016). Cofilin-actin rod formation represents a possible molecular mechanism for the chronic neuroinflammatory hypothesis of AD (Walsh et al., 2014). In all these cases, cofilin disrupts the normal balance of actin dynamics, thus exacerbating the oxidative cascade of neurodegeneration by accelerating mitochondrial decline and ATP depletion (Bernstein et al., 2006; Klamt et al., 2009; Kotiadis et al., 2012). The results presented here positively supports that cofilin might bridge and unite all the hypotheses of AD pathology.

Numerous studies have implicated the dysregulation of cofilin in AD (Bamburg and Bernstein, 2016; Shaw and Bamburg, 2017). It was reported that cofilin protein level was significantly increased in APP transgenic mouse brains and neurons (Yao et al., 2010). A recent study using cofilin immunofluorescence to compare the brains of human AD subjects with those of age-matched controls found that rod-like and aggregate cofilin pathology was four-fold greater in number and larger in area in the brains of AD subjects (Rahman et al., 2014). Studies of Aβ-overproducing mice have shown that decreasing cofilin dephosphorylation or decreasing total levels of cofilin expression are both effective in reducing the cognitive deficits (Woo et al., 2015a,b). However, to the best of our knowledge, there are few relevant studies of cofilin in blood of AD patients.

We confirmed that serum cofilin 2 levels were significantly higher in AD or MCI patients compared to controls. In addition, we observed a strong negative correlation between serum cofilin 2 levels and MMSE scores in AD patients, which suggested that higher cofilin 2 levels were associated with more severe disease. Through ROC curve analysis, we revealed that the sensitivity and specificity were 93 and 87% for serum cofilin 2 greater than 130.4 pg/ml, and its diagnostic accuracy was 80% in identifying AD patients. In addition, we found a significant increase of serum cofilin 2 in AD, and an increased trend but not significant in VaD compared to the controls. Cofilin 2 was previously reported to be significantly increased in protein expressions and phosphorylation levels, which was participated in the pathogenesis of idiopathic dilated cardiomyopathy with amyloid-like aggregates (Subramanian et al., 2015). Thus, it might be an overall marker for these degenerative diseases.

As we have ever known, there are 3 isoforms: cofilin-1, cofilin-2 and destrin, in which cofilin-1 and cofilin-2 have overlapping functions. Interestingly, we found in this study that cofilin 1 was undetectable in serum from both AD patients and controls by western blot. This is the first time to demonstrate the difference of expression of cofilin 1 from cofilin 2 in serum. As for why there is this discrepancy, and the exact roles in AD for cofilin 1 and cofilin 2, respectively, it remains to study further. Though we can’t identify the exact role of cofilin 2 in AD, however, we detected the increased levels of cofilin 2 in human serum during the process. Furthermore, cofilin 2 performed well as a diagnostic and non-invasive biomarker with high sensitivity and specificity. So, it is still meaningful to develop cofilin 2 as a diagnostic biomarker of AD.

In summary, cofilin 2 expression was demonstrated to be significantly increased in AD patients and different AD models (animal and cell) in our present study. The good correlation between MMSE scores and cofilin 2 levels suggests that cofilin 2 might be used to diagnose disease severity. ROC analysis showed that cofilin 2 had high diagnostic values as a reliable biomarker to distinguish patients with AD from healthy subjects. Accordingly, our results highlight potential serum biomarkers of AD, which may facilitate AD diagnosis and assist in the evaluation of anti-AD drugs in both animal models and patients. Further investigation is needed to explore the value of cofilin 2 as a predictor of AD in a larger and independent population of AD and MCI patients.

## Data Availability

The raw data supporting the conclusions of this manuscript will be made available by the authors, without undue reservation, to any qualified researcher.

## Ethics Statement

This study was carried out in accordance with the ethical standards of the Committee of the Affiliated Yuhuangding Hospital of Qingdao University on Human Experimentation of the institution. All subjects gave written informed consent in accordance with the Declaration of Helsinki. The protocol was approved by the Institute Ethical Committee of the Affiliated Yuhuangding Hospital of Qingdao University. This study was carried out in accordance with the institutional guidelines of the Experimental Animal Center of Ludong University. The protocol was approved by the Institutional Review Board of Ludong University.

## Author Contributions

HG and YS conceived and designed the studies. MD enrolled all the subjects and collected the serum samples. YS, LL, and ZL performed the research. CL modified the figures. YS and LL analyzed the data and wrote the manuscript.

## Conflict of Interest Statement

The authors declare that the research was conducted in the absence of any commercial or financial relationships that could be construed as a potential conflict of interest.
